# Additive Manufacturing of Polyether Ether Ketone (PEEK) for Space Applications: A Nanosat Polymeric Structure

**DOI:** 10.3390/polym13010011

**Published:** 2020-12-22

**Authors:** Marianna Rinaldi, Federico Cecchini, Lucia Pigliaru, Tommaso Ghidini, Francesco Lumaca, Francesca Nanni

**Affiliations:** 1 Department of Enterprise Engineering, University of Rome “Tor Vergata”, Via del Politecnico 1, 00133 Rome, Italy; fnanni@ing.uniroma2.it; 2 INSTM—Italian Interuniversity Consortium on Materials Science and Technology, RU Roma Tor Vergata, Via del Politecnico 1, 00133 Rome, Italy; 3 TAS-I Thales Alenia Space Italia, Via Saccomuro, 19-21, 00131 Rome, Italy; federico.cecchini@thalesalenaispace.com (F.C.); francesco.lumaca@thalesaleniaspace.com (F.L.); 4 ESA/ESTEC European Space Agency, NL-2200AG Noordwijk, The Netherlands; lucia.Pigliaru@esa.int (L.P.); tommaso.ghidini@esa.int (T.G.)

**Keywords:** polyether ether ketone, additive manufacturing, PEEK, nanosat, outgassing, fused-deposition modeling, topology optimization, system design

## Abstract

Recent improvements in additive layer manufacturing (ALM) have provided new designs of geometrically complex structures with lighter materials and low processing costs. The use of additive manufacturing in spacecraft production is opening up many new possibilities in both design and fabrication, allowing for the reduction of the weight of the structure subsystems. In this aim, polymeric ALM structures can become a choice, in terms of lightweight and demisability, as far as good thermomechanical properties. Moreover, provided that fused-deposition modeling (FDM) is used, nanosats and other structures could be easily produced in space. However, the choice of the material is a crucial step of the process, as the final performance of the printed parts is strongly dependent on three pillars: design, material, and printing process. As a high-performance technopolymer, polyether ether ketone (PEEK) has been adopted to fabricate parts via ALM; however, the space compatibility of 3D-printed parts remains not demonstrated. This work aimed to realize a nanosat polymeric structure via FDM, including all the phases of the development process: thermomechanical design, raw material selection, printing process tuning, and manufacturing of a proof of concept of a technological model. The design phase includes the application of topology optimization to maximize mass saving and take full advantage of the ALM capability. 3D-printed parts were characterized via thermomechanical tests, outgassing tests of 3D-printed parts are reported confirming the outstanding performance of polyether ether ketone and its potential as a material for structural space application.

## 1. Introduction

Over the last decade, the space industry has seen an increased interest in small satellite missions and the recent advances in commercial-off-the-shelf (COTS) electronic parts miniaturization provided a valuable boost to the development of small spacecraft missions, usually based on nanosat and CubeSat satellites. nanosats are a class of satellites with small dimensions characterized by low weight (between 1 and 10 kilos) and typically used for educational and research project; they are usually referred in terms of CubeSat standard where (1 U is a 1 unit cube sat with a 10 cm^3^ volume and maximum mass of 1 kg) [1].

Nanosats are usually composed of the same subsystems of bigger spacecraft and share the typical subsystems and the relevant design constraints, including the ones on lightweight structure and space environment.

In view of weight reduction, topology optimization has been primarily developed and adopted to design high-performance yet lightweight structures. Topology optimization (TO) methods solve the material distribution problem to generate an optimal topology, but its exploited application is commonly limited by geometrical complexity and manufacturing constraints of conventional fabrication processes [2,3]. The topological optimization process requires a new approach to design to including a new complex structure; it has been used for research in several structural engineering applications [4].

Additive manufacturing (AM) technologies instead, allow building 3D as-designed structures in a layer-by-layer procedure by placing the material only where needed [5,6,7] regardless of the geometrical complexities. AM can easily create a freeform design from topology optimization, eliminating the manufacturing issues and becoming an alternative to conventional fabrication methods [8,9]. The use of AM can lead to an increase in performance in terms of mass savings (between 40% and 70% [10]). Among the other AM techniques, the use of FDM (fused deposition modeling) to produce low-cost CubeSats and nanosats gained importance more recently [11,12,13], as it represents an enabling choice for applied space research and hands-on education.

FDM, or fused-filament fabrication (FFF), is an additive manufacturing process that uses a thermoplastic polymeric material in the form of continuous filament to build parts layer-by-layer.

In [14], an AM-induced evolution of the design process for small satellites was investigated. The aim was to introduce a new concept of spacecraft structural design, capable of taking full advantage of new production technologies. A CubeSat was eventually printed in Polylactic acid (PLA) as a demonstrator. Although this work represents an appreciable innovation, the realized design and FEM analysis lead to a configuration that does not take into account the actual anisotropy of mechanical properties of the 3D-printed structures [15,16,17]. In order to obtain a proper mechanical design suitable for practical application, the numerical simulation and the design should take into account at least factors as the anisotropy of the components (which is the result of the direction of printing of the FDM parts), the interface bonding resistance among adjacent layers and the nonlinear constitutive behavior of the polymer. These factors are proven to be crucial for the optimization of spacecraft and structures as a whole [18,19,20].

In the literature, ABS and PLA are the standards, low-end polymers used for 3D extrusion printing, as they are very easy to process and cheap. Nevertheless, they have limited usefulness when dealing with space systems due to low mechanical properties and space environment limitations (such as outgassing properties, radiation resistance, etc.).

The material’s (polymers) selection for FDM space structures is strongly limited by the space environment and the relevant applicable standards (i.e., ECSS). One of the most critical issues regards the vacuum, which generates material outgassing, defined as the release of gaseous species from the material under high vacuum conditions. The outgassing of materials can be harmful to the performance of a spacecraft for two main reasons: material properties degradation and molecular contamination. In polymers, the nature and extent of outgassing can produce severe changes in the material properties, while contamination can be detrimental for thermal control surfaces and the payload altering the performance of optical instruments (condensed outgassing products may obscure surfaces), instruments (readings can be affected by local clouds) and solar cells on-board the spacecraft [21,22,23]. According to European Space Agengy (ESA) standards (ECSS-Q-70), only very few polymers are suitable for use in space. Among the few, technopolymers (as polyether ether ketone (PEEK) and polyetherimide (PEI)) offer the highest mechanical properties [24,25] while meeting the outgassing requirements and sustaining the harsh conditions characterizing outer space. VICTREX^®^ PEEK is even listed in the low outgassing section of NASA’s outgassing data for selecting spacecraft materials, and the material has seen extensive use in space flight applications [26].

PEEK is a semicrystalline thermoplastic polymer with superior mechanical, thermal and chemical properties. Injection-molded PEEK exhibits an elastic modulus of around 3.6 GPa and ultimate strength of 100 MPa [27]. PEEK offers high-temperature and chemical stability, as it can be dissolved only at high temperatures in sulfuric acid [28].

The use of PEEK as thermoplastic feedstock materials for fused deposition modeling (FDM) has been reported by several papers [29,30], mostly related to space and biomedical applications. However, the outgassing properties of PEEK samples produced via FDM have never been tested. NASA maintains a database of material outgassing properties; PEEK 3D-printed actual values are not available. It could be argued that the material may “bake-out” during the extrusion process lowering the mass loss value, but the FDM process can be detrimental as well since 3d printed samples have been demonstrated to have an intrinsic porosity [31].

In this article, the design for additive manufacturing (DFAM) approach was applied to realize a PEEK-FDM-printed nanosat. The AM-oriented thermomechanical design, based on topology optimization and multistep approach, was carried out focusing on the use of a thermoplastic material: therefore, addressing the issues concerning both the mechanical design and the thermal performance (need to dissipate the heat generated by the payloads). A specific low-Earth orbital mission profile was considered as design input. The nanosat design was carried out with an iterative procedure [32] to harmonize all the thermal and mechanical issues, as the functionality of the whole depends on the synergic optimization of each step. Further details about the design procedure are reported in the “Materials and methods“ section.

The knowledge of the 3D-printed parts mechanical and thermal behaviors is of tremendous interest to properly design structural elements; however, the datasheet available are mostly referred to technical specifications of standard filaments and injection-molded parts. In this sense, some very recent publications address the issue, providing both the materials properties and the correlation with numerical simulation [33,34].

To verify the design hypothesis, 3D-printed PEEK specimens were prepared and characterized in terms of thermal, thermomechanical and mechanical tests (dynamic mechanical thermal analysis (DMTA), tensile and flexural) and outgassing performance as per the ESA standards [35].

The experimental phase of this work is composed of three main activities:

- Nanosat platform thermomechanical design (mission design hypothesis, DFAM (design for additive manufacturing) and topology optimization);
- FDM printing and characterization of PEEK laboratory specimens printed by FDM, to verify the compliance of the performance of printed parts capability to achieve the mechanical properties to those assumed in the design (mechanical, thermal, and to verify the outgassing) requirements;
- FDM printing of a representative PEEK nanosat.

## 2. Materials and Methods

### 2.1. Nanosat Thermomechanical Design

At first, the mission requirements were identified, assuming the nanosat to operate in low Earth orbit (LEO) with an altitude ranging between 400 and 600 km. The design and dimensioning of the spacecraft primary structure are given by the launch vehicle user manual (Soyuz launch vehicle) [36].

Table 1 summarizes the first five frequencies considered as design constrains:

The design and dimensioning of the spacecraft primary structure with the Soyuz launch vehicle system shall be based on the design load factors. The design load factors are represented by the Quasi-static-loads (QSL) that are the more severe combinations of dynamic and steady-state accelerations that can be encountered at any instant of the mission (ground and flight operations). Moreover, it is necessary to introduce safety factors, commonly used in space projects, to account for uncertainties regarding the prediction of loads, structural analysis, the fabrication process and material properties. In this case, to make the design safer:

- A coefficient based on the model and owed by its uncertainty in making a hypothesis to proceed in design: $X_{m}=1.1;$
- A coefficient based on design and environment to avoid risk during the design and the test phase (which takes into account the differences between the filament and 3D-printed parts): $X_{d}=1.25;$

The resultant coefficient, which is used in the analysis to make indeed the design safer, results to be $X_{s}=X_{m}*\;X_{d}=1.375$. The final values of QSL are reported in Table 2 and are used to pre-multiply the loads that occur in the ascent profile of the launch vehicle system.

An equivalent σ, based on the von Mises criterion: $\sigma_{\max}\;$< 45.45 MPa (the upper bound is given from $\frac{\sigma_{y}}{X_{m}}$ = $\frac{50}{1.1}$) was applied.

The nanosat structure is a 2 U CubeSat, with a total occupied volume of 10 × 10 × 21 cm (including the payload). The nanosat model was discretized using Hyperworks 13.0 suite by Altair Engineering: structure properties and boundary conditions were set with Hypermesh and the optimization of the geometry with Optistruct. The optimization results were post-processed with Hyperview.

**Figure 1:**
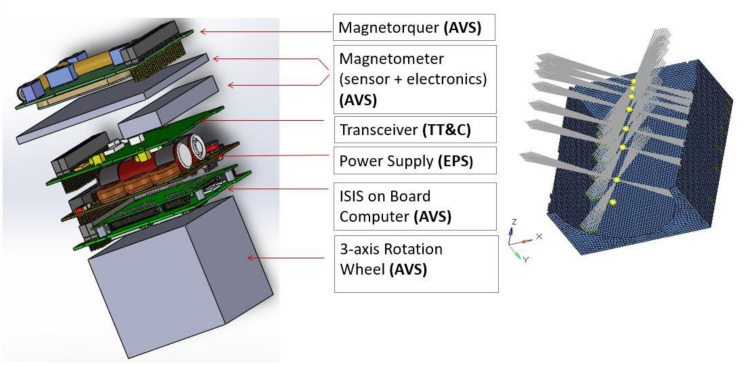
(left) Rendering of internal hardware; (right) Platform structure and internal hardware (equipment/payload) as concentrated Masses (yellow points).

The thermal analysis of the nanosat structure was carried out with Nastran. The QSL factors apply to equipment and payload (Centre of Gravity) CoG (internal boards are modeled with lumped masses). Figure 1 depicts the inner equipment distribution: dimensions, mass, power, and CoG are reported in Table 3.

The thermal loads were distinguished in two contributes:

- Internal heat: the power generated by all the internal devices (listed in Table 3), considering all the devices active simultaneously. The total power is dissipated in 4 different spots (joints between board and structure). Internal radiation was considered negligible, and a conservative temperature T_joint_ < 60 °C was imposed as a constraint [37]. The analysis was carried out as a nonlinear study, iteratively calculated by the solver Nastran.
- External heat: radiative [38] fluxes coming from the space were neglected, as this heat was supposed to be managed by adding an external wrapping foil of thermal protection.

To complete the thermal analysis, we assumed a constant temperature of 4 K of the environment, with a view factor of 1.0 for each surface and surface emissivity of 0.8 (gray body).

Finite element method (FEM) analysis was carried out considering PEEK as material for the nanosat production. The constitutive behavior of the material was considered linear-elastic, isotropic, and homogeneous. The material properties used as input of the FEM were taken at first from the datasheet. Therefore, a corrective factor based on the experimental results obtained by testing the AM printed laboratory samples (see Section 3.2) was applied where needed/appropriated.

The data used in the simulation are reported in Table 4.

**Figure 2:**
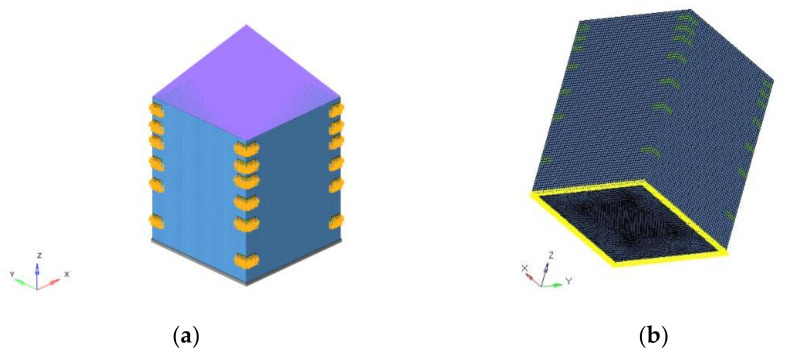
Picture of the bounding box and mesh used for the topology optimization (**a**) top view (**b**) bottom view.

Surface discretization was used with “quadrilateral mesh” (e.g., CQUAD4) [6]. The realized mesh (detailed reported in Table 5) is formed by 42,520 elements (PSHELL) with a global element dimension of 3 mm on average. All internal devices were considered concentrated masses at grid points (Figure 1), while the payload is a concentrated mass outside the structure. It was assumed that any device is constrained to the structure in four anchoring points. The lumped masses were connected to the structure through a 1D element (RBE3), linking two different grid points. The bounding box is reported in Figure 2; the purple lines indicated the link with the payload.

The optimization was focused on minimizing the nanosat total design-mass to achieve the best minimization of this parameter (the absolute minimum of the function). Two different optimization methods were adopted at the same time: topological and topometric analysis (the variables of these analyses are the properties given to the mesh of the model). Topology optimization methods solve a material distribution problem to generate optimal topology. Each finite element within the design domain was defined as a design variable, allowing a density variation (SIMP approach). The topology optimization result is a “density distribution” of the finite elements in the design domain. The design variable in this method is the normalized material density of each element, which is defined as:

(1) $$\rho_{i}=\rho_{ai}/\rho_{0}$$

where *ρ_i_* is the normalized material density of the element *i*, *ρ_ai_* is the assumed material density of the element *i*, and *ρ*_0_ is the actual material density.

Analogously, the thickness of each element was set as the variable of the topometric analysis (on each PSHELL element). This allowed finding the optimal thickness (the minimum) that still respects the given constraints requested by the design loads.

To take into account both the static and the modal response of the structure, the optimization was carried out using a “combined compliance index”. The “compliance” [8,40] is defined as the structure’s strain energy and can be considered a reciprocal measure for the stiffness of the structure. The compliance was implemented through an equivalent sum (“compliance index”) to comprise the performed dynamic analysis. The “combined compliance index” includes multiple frequencies and static subcases (load steps, load cases) combined in the topology optimization. The index was defined as follows:

(2) $$S=\;\Sigma w_{i}\cdot C_{i}+NORM\;\frac{\Sigma w_{j}\cdot I\lambda_{j}}{\Sigma w_{j}}$$

that is a global response defined for the whole structure [41,42].

Where *C_i_* are the compliance values, *λ_j_* are the eigenvalues, *w_i_*_,_ and *w_j_* the weights for each load case for the optimization procedure. The normalized factor, NORM, is used for normalizing the contributions of compliances and eigenvalues. A typical structural compliance value is of the order of $10^{-4}$ to $10^{-6}$. However, a typical inverse eigenvalue is on the order of $10^{-5}$. If NORM is not used, the linear static compliance requirements dominate the solution. Due to this consideration, the quantity NORM is typically computed using the formula:

$$NF=\;C_{max}\cdot \;\lambda_{min},$$

where $C_{max}$ is the highest compliance value in all subcases (load steps, load cases) and $\lambda_{min}$ is the lowest eigenvalue included in the index. The NORM value can be arbitrarily imposed. Otherwise, it is automatically calculated by the solver [13].

### 2.2. 3D Printing of PEEK Samples

**Figure 3:**
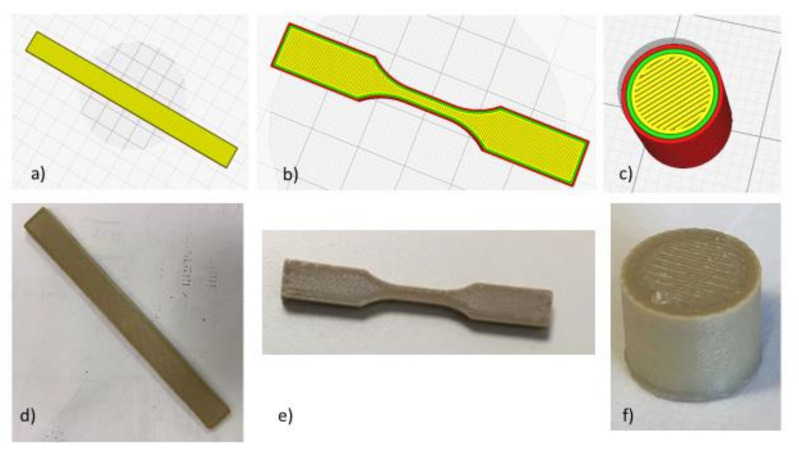
(**a**,**d**) CAD (upper) and photo (lower) of 3D-printed flexural test specimen; (**b**,**e**) tensile test specimen; (**c**,**f**) outgassing specimen.

Tensile specimens (configuration of specimens in line with ASTM D638 Type V micro-tensile specimens, length = 63.5 mm, width = 9.53 mm and thickness = 3 mm) [43]. Flexural testing specimens (dimensions according to the ASTM D790:127 mm long × 12.7 mm large × 3.2 mm-thick) and Outgassing test samples (diameter 60 mm, height 20 mm, 3 specimens produced and tested) were printed horizontally on a building platform plane (XY), with a raster angle of +45°/−45° in alternate layers, layer height of 0.2 mm. Two perimeters were set as contour width Z. The specimens (Figure 3) will be referred to in the following as PEEK_XY_flex (bending test specimens Figure 3a,d), PEEK_XY_tens (tensile test specimens Figure 3b,e), and PEEK_3D_outgas (outgassing specimens Figure 3c,f). The printing parameters are reported in Table 6.

The CAD drawings of each type of specimen were imported into the Simplify 3D software to set the slicing and the printing parameters. An APIUM FDM 3D printer was used with PEEK filament (purchased from APIUM) printing, using the same parameter set up used in another previous work [29]. The tensile test was performed with a Zwick/Roell Z100 load frame (load cell of 2.5 kN, @1 mm/min, equipped with a laser extensometer) on five specimens.

Three-point bending tests were carried out on five specimens by using an Instron 5569 universal testing machine, according to ASTM D790 (crosshead movement 1.45 mm/min.; Span Length 54.4 mm). Density measurements (Sartorius) were carried out on PEEK_XY_tens and PEEK_3D_outgas specimens. Scanning electron microscopy analysis (FE-SEM, Cambridge Leo Supra 35) of gold-sputtered (25 mA, 1 × 10^−4^ Bar, 120 s) cryo-fractured PEEK_XY_tens specimens was carried out too.

Thermomechanical analysis (Triton DMTA Tensile mode Temperature range −50:250 °C @1 Hz 0.05) was conducted to evaluate the storage modulus at 25 °C and the Tg of printed material. Tests replied in triplicates. Outgassing test was performed in accordance with [35] in the μVCM facility at ESA/ESTEC laboratory in the Netherlands.

The procedure consists of the following steps:

⮚ Pretest conditioning of the sample, 100–300 mg of material (unless mentioned otherwise), was 24 h at 22 ± 3 °C and 55 ± 10% RH.
⮚ During the test for 24 h:
  ✔ The sample was subjected to a temperature of 125 °C;
  ✔ The condensable material was collected by a collector plate kept at 25 °C;
  ✔ The test vacuum pressure was kept below 10^−5^ mbar.
⮚ Posttest conditioning of the sample was 24 h at 22 ± 3 °C and 55 ± 10% RH [44].

The relevant outgassing parameters measured during the test and their definitions are listed in the following:

- RML (recovered mass loss): total mass loss of the specimen itself without absorbed water. RML is introduced because water is not always seen as a critical contaminant in the spacecraft. Generally, the equation holds RML = TML-WVR;
- TML (total mass loss): total mass loss of the material outgassed from a specimen that is maintained at a specific constant temperature and operating for a specific time. TML is calculated from the specimen mass as measured before and after the test and is expressed as a percentage of the initial specimen mass;
- WVR (water vapor regained are measured): the mass of the water vapor regained by the specimen after the optional reconditioning step;
- CVCM (collected volatile condensable material): quantity of outgassed matter from a test specimen that condenses on a collector maintained at a specific temperature for a specific time. CVCM is expressed as a percentage of the initial specimen mass. It is calculated from the condensate mass determined from the difference in the collector plate’s mass before and after the test.

**Figure 4:**
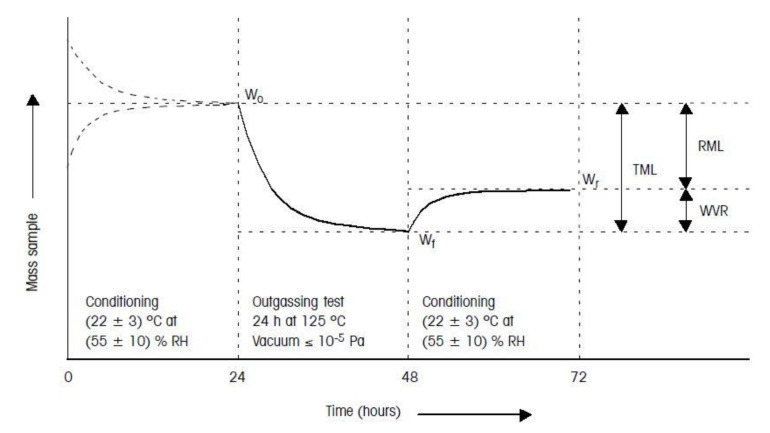
Outgassing test sequence according to ECSS standard [35]. The mass sample is plotted versus time.

The test sequence procedure (as per ECSS standard [35]) is sketched in Figure 4.

According to ECSS-Q-ST-70-02C standard [35], space-qualified materials should have as a minimum: RML < 1.0%, CVCM < 0.10%. The acceptance limits can be more stringent for materials used in the fabrication of optical devices or in their vicinity.

## 3. Results and Discussion

### 3.1. Numerical Analysis Results

**Figure 5:**
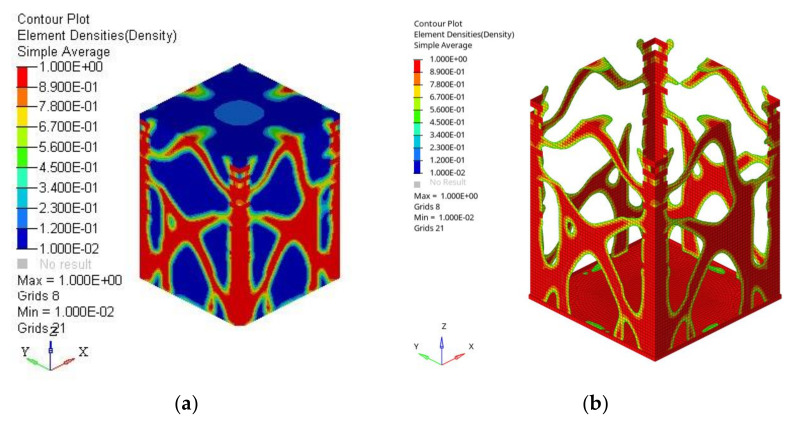
(**a**) Contour plot of topological optimization: the red elements form the optimized structure output (normalized material density ρ_i_ = 1), the blue elements (normalized material density ρ_i_ = 0) are negligible; hence not part of the final optimized structure. (**b**) final mesh obtained after the optimization process.

The contour plot reported in Figure 5a shows the topological optimization results in terms of ρ_i_ (Equation (1)). The design variable ρ_i_ may vary between 0 and 1. If the normalized material density is close to 0, the element is considered negligible by the optimization analysis (blue parts). On the other hand, if the ρ_i_ of an element is close to 1 (red areas), the element should exist in the optimal topology and be part of the final optimized structure, as shown in Figure 5b.

The output of the topological optimization was then modified and completed by integrating the thermal analysis.

In the process of substituting metal with polymers in a primary structure, despite the compliance of the mechanical design, other issues may arise; in addition to the mechanical requirements is necessary to consider the material thermal conductivity and the ability of the structure to dissipate the heat produced by the internal equipment.

As previously reported, the main thermal contribution was assumed to be the heat generated by the electronic equipment that, while negligible in terms of radiation, is relevant in terms of conductance. This latter parameter, usually neglected when working with metals (conductivity K in the order > 100 W/mK), becomes an issue when polymers are involved, as they are bad thermal conductors [45]. Moreover, the low heat dissipation can cause detrimental structural issues as the temperature approaches the polymer Tg (glass transition temperature).

**Figure 6:**
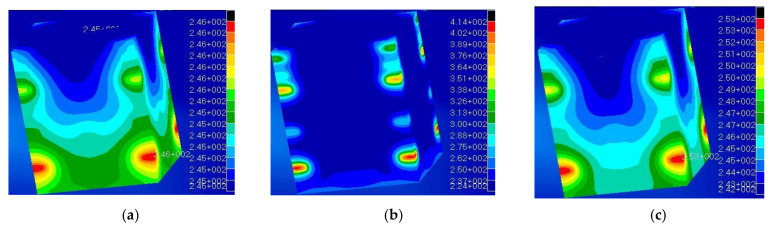
(**a**) Contour of temperature with aluminum (K = 170 W/mK); (**b**) contour of temperature with PEEK (K = 0.2 W/mK); (**c**) identified requested threshold value K = 5 W/mK to reach satisfactory heat dissipation.

Figure 6 reports the results of the thermal analysis. The contour plot is referred to the local temperature reached considering the actual thermal conductivity of aluminum (Figure 6a) and PEEK (Figure 6b). PEEK offers a thermal conductivity of K = 0.25 W/mK [46], which is 3 orders of magnitude lower than that of the metals used in aerospace applications, i.e., aluminum (KALUM = 170 W/mK). The reduced thermal conductivity leads the polymer to reach high temperatures (red spots reaching 414 K) concentrated in small areas where the electronic equipment is placed. Those temperatures are higher than the polymer’s glass transition temperature, thus producing a local reduction in the material stiffness, possibly leading to significant thermal distortions and nonnegligible deformations, which may compromise the structural integrity of the nanosat itself. In Figure 6a, the maximum temperature (red elements) is 246 K while the minimum is 245 K, indicating and effective heat dissipation. Figure 6b instead clearly shows how the PEEK structure presents high-temperature gradients, from the red spots at 414 K to the blue zones at 224 K.

This analysis makes clear that heat management is a crucial issue when designing polymer structures. In particular, the results of a subsequent analysis lead to identifying a minimum target value for the polymer thermal conductivity, which would allow overcoming the thermal dissipation issues. According to existing literature [47,48], a satisfactory maximum temperature difference of 40 °C between the inner and outer layers was fixed, leading to an acceptable value (for a feasible design) of K = 5 W/mK (contour plot in Figure 6c). This value could be achieved either using bespoken polymeric composite materials [49,50,51,52] or adopting a heat sink and protective layers and coatings (MLI, white and black paint) [53].

### 3.2. Material Characterization

The mechanical properties of PEEK printed specimens tested are reported in Table 7. The mechanical properties of PEEK obtained from tensile and flexural tests were compared with the material datasheet (manufactured via injection molding). The value of experimental tensile and flexural modulus, as well as the ultimate strength, are lower than expected compared to the traditional manufacturing process; however, the results are in line with literature on 3D-printed PEEK [54,55].

**Figure 7:**
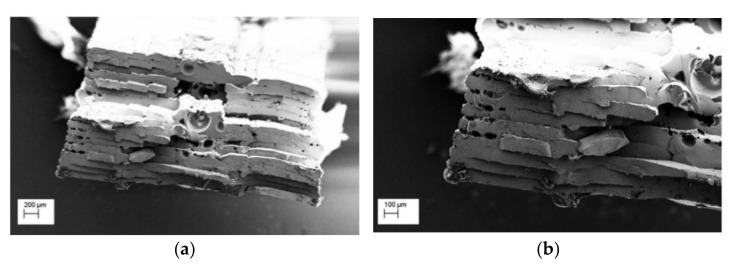
SEM micrograph of 3D-printed PEEK tensile specimen (**a**) low magnification (**b**) high magnification.

The results of the density measurements of 3D-printed specimens are reported in Table 8. Compared to the bulk material nominal value, as for the datasheet, it can be concluded that the 3D-printed parts are not fully dense. The SEM analysis (Figure 7) confirms these results, as some porosity was observed in the center of cryo-fractured tensile test specimens. This could affect the outgassing properties, as the presence of voids may influence the results in terms of RML (recovered mass loss) and CVCM.

**Figure 8:**
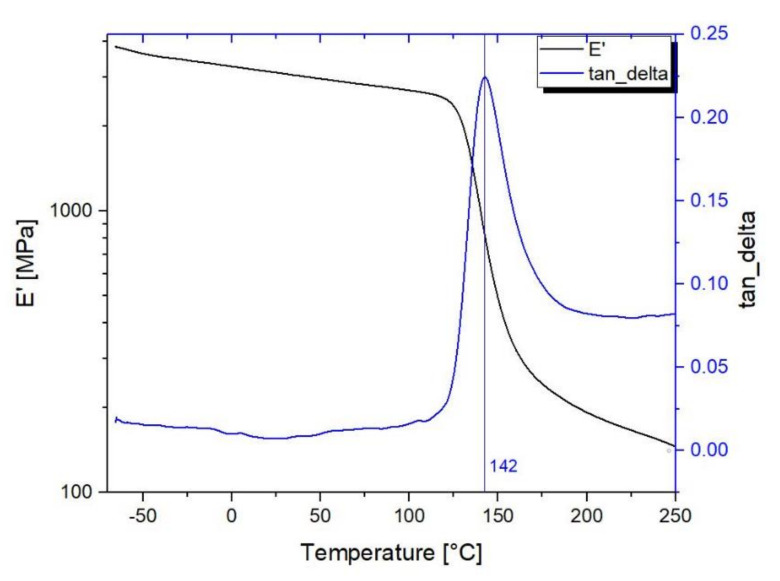
DMTA storage modulus E’ of 3D-printed PEEK as a function of temperature.

In Figure 8, the dynamic-mechanical properties related to the elastic storage modulus (E′), loss modulus (E″ and mechanical damping tanδ curves are presented for a typical DMTA scan for 3D-printed PEEK samples.

DMTA results confirm that the value of the storage modulus (E’) is relatively stable in the typical operational range (−70 + 120) [56], varying from 3.9 GPa at −70 °C to 2.3 GPa at 125 °C, where the E’ presents the onset of decrement and at it remains above 1 GPa up to 140 °C. The high value of storage modulus is almost constant in the operational temperature range and entirely in line with the nanosat operational environment. The value of glass transition temperature as the peak of tanδ is at 153 °C, in line with the scientific literature [29,31].

### 3.3. Outgassing

The results of the outgassing tests are reported in Table 9.

Polymers, in particular in their amorphous structures, can dissolve significant quantities of gas; the relatively high mobility of the gas through the polymeric chains results in the outgassing rates [57,58].

In our case, the outgassing requirements of 3D-printed parts are fully satisfied, being the RML far below the maximum acceptable value (average value of 0.11%) and CVCM ten times below the maximum acceptable value, as from the ECSS. This result can be expected, as PEEK is highly semicrystalline [58]. Moreover, a comparison between our 3D-printed PEEK part and the NASA OUTGASSING database for PEEK 450 pellets demonstrates that the 3D printing does not affect the PEEK outgassing properties.

### 3.4. 3D Printing of the PEEK Nanosat

**Figure 9:**
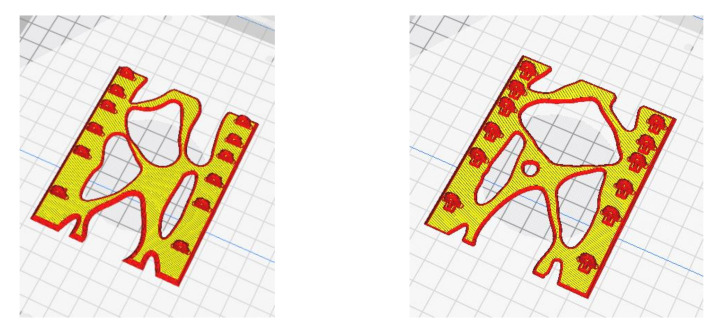
Nanosat optimized (**left**) and (**right**) side structures.

At the end of the work, having updated the TO of the nanosat in view of the experimental results achieved during the characterization campaign on 3D-printed PEEK, an optimized design of the PEEK nanosat structure was accomplished. Each face of the PEEK nanosat was designed following the density plot (results Section 3.1), then the CAD part was sliced, and process parameters were set via Simplify 3D (Figure 9) and printed via FDM.

It should be noted that the final design is strongly asymmetrical to adapt to the actual stress distribution, which is not symmetric, as a result of the CoG distribution of equipment. Thanks to the FDM, 3D printing of topologically optimized structure was possible, realizing the fully optimized structure and saving weight.

**Figure 10:**
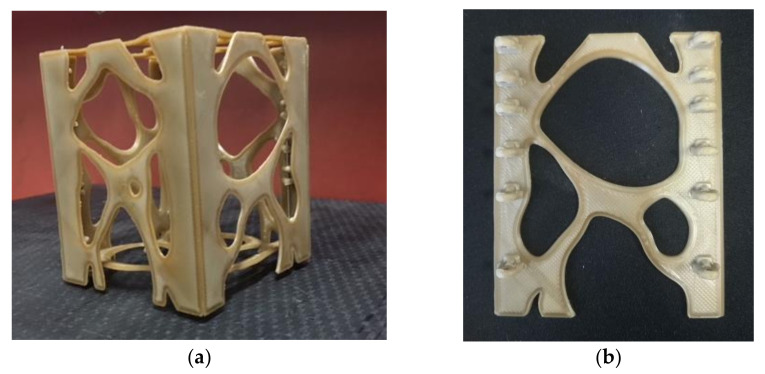
3D-printed PEEK optimized nanosat. (**a**) view of the entire 3D printed Nanosat (**b**) detail of one 3D printed side structure.

Figure 10 reports a picture of the printed nanosat. All faces were printed in the XY plane to maximize the mechanical properties of the parts and then assembled. A single printing job for the entire nanosat could have been used; nevertheless, much lower mechanical properties would have been expected [16,59].

## 4. Conclusions

In this study, the use of PEEK as a structural material for space application was investigated, and a PEEK nanosat structure was 3D-printed as a proof of concept. The research started with the thermomechanical design and relevant topological and topometric optimization, then the effect of the 3D printing process on the material was assessed via experimental characterization. The mechanical and thermal analysis confirmed that the printing process slightly affects the material mechanical properties, while the thermal properties remain unaffected.

Density measurement confirmed the internal porosity of 3D-printed parts as an unavoidable effect of the FDM process; however, the outgassing of 3D-printed PEEK was confirmed to be very low and in line with the standard requirements to be used for space application.

A PEEK nanosat was then printed accordingly, demonstrating the feasibility of using high-performance ECSS compliant polymers in the production of space components via ALM. Although this target was reached, the thermal issue remains a major open point. The thermomechanical design evidenced that a minimum value of thermal conductivity (i.e., 5 W/mK) should be ensured to avoid the detrimental effects of “hot spots” generated by the internal devices. Such value is much above that showed by neat polymers. In particular, the temperature shall not exceed the polymer glass transition temperature to avoid the mechanical properties drop (particularly in terms of stiffness).

## Figures and Tables

**Table 1:** Design constrain frequencies.

Lower Limit of Allowable Frequencies	
I frequency	>50 Hz
II frequency	>51 Hz
III frequency	>70 Hz
IV frequency	>90 Hz
V frequency	>110 Hz

**Table 2:** Quasi-static loads (including safety factors).

	Total QSL Resultant (with Safety Factor)	
**Ground and Flight Load Cases**	Lateral	Longitudinal
	±4675 g	−7.9 g/+4.95 g

**Table 3:** Hardware matrix.

Equipment	Dimension (mm)	Mass (kg)	Power (W)	Disposition (z COG, mm)
Mai-201 3-axis wheel	76.2 × 76.2 × 70	0.64	2.2	35
On-board computer	96 × 90 × 12.4	0.094	0.4	86.2
Power supply	96 × 90 × 26	0.2	0.025	115.4
Transceiver	96 × 90 × 15	0.085	1.7	145.9
Magnetometer (electronics)	90 × 30 × 11	0.15	0.4	165.9
Magnometer (sensor)	100 × 100 × 5	0.015	0	178.9
Magnetorquer board	95 × 90.1 × 15	0.195	0.96	190
Payload	100 × 100 × 50	3	0	225

**Table 4:** Materials properties referred to injection-molded parts.

	PEEK [39]
Tensile modulus E (GPa)	4
Flexural modulus (GPa)	165
$\sigma_{y}$ (MPa)	90
Tg (°C)	143
Tm(°C)	343
K(W/mK)	0.25
ρ (kg/$m^{3})$	1300

**Table 5:** Mesh details.

Type of Elements	PSHELL
Number of elements	42,520
Elements size	3 mm
Number of nodes	38,548
Mesh quality	worst aspect ratio: 1.7 (admissible < 5, 0% failed) worst warpage = 0 (admissible < 15, 0% failed) worst skew = 29.7 (admissible < 40, 0% failed) worst Jacobian = 0.63 (admissible > 0.6, 0% failed) worst taper = 0.39 (admissible < 0.6, 0% failed) max quad angles = 135° (admissible < 140°, 0% failed) min quad angles = 53.2° (admissible > 40°, 0% failed) max tria angles = 77.6° (admissible < 120°, 0% failed) min tria angles = 33.8° (admissible > 30°, 0% failed)

**Table 6:** 3D printing parameters.

3D Printing Parameters	Value
Nozzle diameter	0.4 mm
Nozzle temperature	410 °C
Bed temperature	120 °C
Layer height	0.2 mm (first layer 0.3 mm)
Printing speed	2000 mm/min
Perimeter	2
Raster angle	+45/−45
Infill density	100%

**Table 7:** Main results of mechanical characterization.

	Max Stress (MPa)	Flexural Modulus (GPa)	Strain (%)
PEEK Datasheet	165	3.6	
PEEK_XY_ flex	173 ± 8	2.85 ± 0.1	1.0 ± 0.1
	**UTS** **(MPa)**	**Tensile Modulus** **(GPa)**	**Elongation at break (%)**
PEEK_Datasheet	98	4	45
PEEK_XY_tens	78.0 ± 7.2	2.9 ± 0.3	6.9 ± 3.0

**Table 8:** Density measurements.

	Density (g/cm^3^) from Datasheet	Density (g/cm^3^) Measured
PEEK 3D Tensile sample	1.300	1.221 ± 0.036
PEEK 3D Outgassing sample	1.300	1.244 ± 0.025

**Table 9:** Outgassing Test Results according to ECSS standard [45].

PEEK_3D	TML (%)	CVCM (%)	RML (%)
Sample 1	0.187	0.01	0.113
Sample 2	0.186	0.01	0.103
Sample 3	0.185	0.01	0.17
Average	0.19	0.01	0.11
Standard deviation	0.00	0.00	0.01
**PEEK 450**	**TML** **(%)**	**CVCM** **(%)**	**RML** **(%)**
NASA OUTGASSING MATERIAL DATABASE	0.3	0.02	0.12

## Data Availability

The data presented in this study are available on request from the corresponding author.
